# Midazolam Indications and Dosing in Palliative Medicine: Results from a Multinational Survey

**DOI:** 10.3390/curroncol31070305

**Published:** 2024-07-19

**Authors:** Morten Tranung, Tora Skeidsvoll Solheim, Erik Torbjørn Løhre, Kristoffer Marsaa, Dagny Faksvåg Haugen, Barry Laird, Morten Thronæs, Michael Due Larsen

**Affiliations:** 1 Department of Clinical and Molecular Medicine, Faculty of Medicine and Health Sciences, Norwegian University of Science and Technology, 7034 Trondheim, Norway; tora.s.solheim@ntnu.no (T.S.S.); erik.t.lohre@ntnu.no (E.T.L.); morten.thrones@ntnu.no (M.T.); michael.d.larsen@ntnu.no (M.D.L.); 2 Department of Clinical Pharmacy, Trondheim Hospital Pharmacy, 7030 Trondheim, Norway; 3 Cancer Clinic, Trondheim University Hospital, St. Olavs Hospital, 7030 Trondheim, Norway; 4 Centre for Crisis Psychology, Faculty of Psychology, University of Bergen, 5006 Bergen, Norway; 5 Department of Multidisease, Copenhagen University Hospital—North Zealand, 3400 Hilleroed, Denmark; dokmarsaa@gmail.com; 6 Regional Centre of Excellence for Palliative Care, Western Norway, Haukeland University Hospital, 5009 Bergen, Norway; dagny.renata.faksvag.haugen@helse-bergen.no; 7 Department of Clinical Medicine K1, University of Bergen, 5007 Bergen, Norway; 8 Institute of Genetics and Cancer, University of Edinburgh, Edinburgh EH8 9YL, UK; barrylaird@ed.ac.uk; 9 Centre for Clinical Epidemiology, Odense University Hospital, 5000 Odense, Denmark

**Keywords:** midazolam, palliative care, palliative medicine, survey, anxiety, dyspnoea

## Abstract

Despite sparse evidence and limited guidance on indications, use, and dosing, midazolam is widely used in palliative care. We aimed to describe and compare the use of midazolam in three different countries to improve clinical practice in palliative care. We performed an online survey among palliative care physicians in Norway, Denmark, and the United Kingdom (UK). The focus was indications, dosing, administration, and concomitant drugs. A web-based questionnaire was distributed to members of the respective national palliative medicine associations. The total response rate was 9.4%. Practices in the UK, Norway, and Denmark were overall similar regarding the indications of midazolam for anxiety, dyspnoea, and pain treatment in combination with opioids. However, physicians in the UK used a higher starting dose for anxiety, dyspnoea, and pain treatment compared to Norway and Denmark, as well as a higher maximum dose. Danish physicians preferred, to a higher degree, on-demand midazolam administration. Despite practice similarities in the UK, Norway, and Denmark, differences exist for midazolam dosing and administration in palliative medicine. We demonstrated a lack of consensus on how midazolam should be used in palliative care, setting the stage for future studies on the topic.

## 1. Introduction

In spite of prolonged survival for several tumour types, cancer is an incurable disease for many patients [1,2]. Towards the end of life, patients with cancer often experience a diversity of burdensome symptoms [3]. Adequate palliative care interventions are essential for good symptom management [4,5].

A systematic review on symptom burden in patients with incurable cancer found that fatigue, pain, loss of appetite, dry mouth, and depressed mood were the five most prevalent symptoms [4]. Other common symptoms included worrying, insomnia, dyspnoea, and anxiety [4]. Anxiety prevalence was estimated to be approximately 30% and unchanged throughout the disease trajectory [4]. A Cochrane review found no high-quality evidence to support drug therapy for anxiety in adult patients receiving palliative care [6]. The review stated that clinical guidelines primarily endorse non-pharmacological interventions and that drugs should be dosed restrictively. Based on the available knowledge, the recommended drugs for anxiety in palliative care patients are short-acting benzodiazepines, such as lorazepam and midazolam [6]. A review from 2017 found one RCT supporting midazolam in combination with morphine over oxygen as a treatment for anxiety [7].

Midazolam is widely used in palliative care [8,9]. Notwithstanding it being considered an essential drug [10,11], few European countries have national guidelines describing the use of midazolam in palliative care. In addition, dosing and treatment practices vary between centres and countries [8,9], and the use of midazolam in palliative care is off-label [12]. And even though low doses may be futile and high doses cause unintended sedation, hypotension, cognitive impairment, amnesia, and respiratory depression, potentially inappropriate midazolam use has not been systematically addressed [8]. 

In palliative care, the anxiolytic and sedative effects of midazolam are often utilized. These effects are mediated through the inhibitory neurotransmitter gamma-aminobutyric acid [9]. Even though benzodiazepines are the drug class of choice when treating acute anxiety in palliative care [13], pharmacological treatment of anxiety in palliative care should be limited to the acute phase [14]. Midazolam can also be utilized to treat agitation and restlessness [9]. In addition, midazolam may be beneficial in the treatment of dyspnoea [15,16]; its role, however, is debated [17]. Furthermore, midazolam can potentially serve as a secondary treatment option for intractable hiccups in palliative care [18].

More importantly, midazolam has anticonvulsant and muscle-relaxant properties and is considered one of the main treatment options for ongoing seizures in palliative care [19]. For status epilepticus, midazolam may also be used after initial symptom control is achieved, making it a convenient drug for refractory seizures [19]. The doses recommended for the treatment of status epilepticus are quite high compared to other indications and can reach levels used for palliative sedation. In fact, midazolam is often utilized for palliative sedation, and European guidelines recommend the drug for this purpose [20]. The dosages used for palliative sedation often supersede the dosages employed for other indications [9].

Its short half-life and rapid onset of action facilitate safe patient monitoring [9], and midazolam may be co-administered with other drugs such as opioids, both on demand (PRN) and as a continuous subcutaneous infusion (CSCI) [21]. 

Due to limited and inconsistent evidence and the widespread use of midazolam, collecting information on the use of midazolam in palliative care in different countries is important. As the first step on the road to improved and standardized use of midazolam in symptom management, we aimed to compare practices in Norway (NO), Denmark (DK), and the United Kingdom (UK).

## 2. Materials and Methods

We performed an online cross-sectional survey among palliative care physicians in NO, DK, and the UK. A working group that comprised authors from the three collaborating countries organized the development and execution of the survey. The study was designed, conducted, and reported in accordance with the Checklist for Reporting Results of Internet E-Surveys (CHERRIES) [22]. 

### 2.1. Participants

Physicians working in palliative care in NO, DK, and the UK were eligible for the survey. Participants were recruited through the respective national palliative medicine associations: Norsk Forening for Palliativ Medisin (NFPM), Dansk Selskab for Palliativ Medicin (DSPaM), and the Association for Palliative Medicine of Great Britain (APM). Only UK members of the APM received the invitation. 

### 2.2. Questionnaire

Based on a review of the literature, we designed a questionnaire on the use of midazolam in palliative care [9,23,24]. The questionnaire was piloted and thereafter updated for functionality, usability, clinical relevance, and reliability. The final questionnaire contained a minimum of 21 questions and was expanded dependent on the number of midazolam indications checked for. The survey included questions on participant demographics, midazolam indications, dosing, administration, drug combinations, treatment duration and withdrawal, and midazolam treatment after discharge. Responses on demographics and on midazolam indications were compulsory. There was a combination of single-response items and multiple-response items, and we opened up the survey for supplementary free-text responses on indications, dosing, and adverse effects. A 5-point Likert scale was used when appropriate.

### 2.3. Data Collection

The questionnaire was set up through an online solution made available by the University of Oslo, Norway [25]. An invitation email with a brief description of the survey, anticipated completion time expenditure, and a link to the online questionnaire was sent to members of NFPM, DSPaM, and APM. The survey was open and voluntary, and all email recipients had access to the questionnaire. To control the target population, invitation recipients were asked not to forward the email. The survey was accessible in February and March 2022 and closed after two reminders.

### 2.4. Analyses

The data were analysed using descriptive statistics and displayed as frequencies and percentages. Medians and the interquartile range (IQR) were used as data and were not distributed normally. 

### 2.5. Ethics

No personal data, email addresses, or Internet Protocol (IP) addresses were registered. The study was beyond the scope of the Norwegian Health Research Act, and approval from the Regional Committee for Medical and Health Research Ethics was not requested [26].

## 3. Results

### 3.1. Participants

Altogether, 126 (9.4%) of the 1338 email recipients responded. There were national differences in response rates (NO 13%, DK 18%, UK 6%). There were no overdue submissions. Three-quarters of the participants were specialists in palliative medicine, and half the participants had worked in palliative care for more than 10 years (Table 1).

### 3.2. Midazolam Indications 

**Figure 1:**
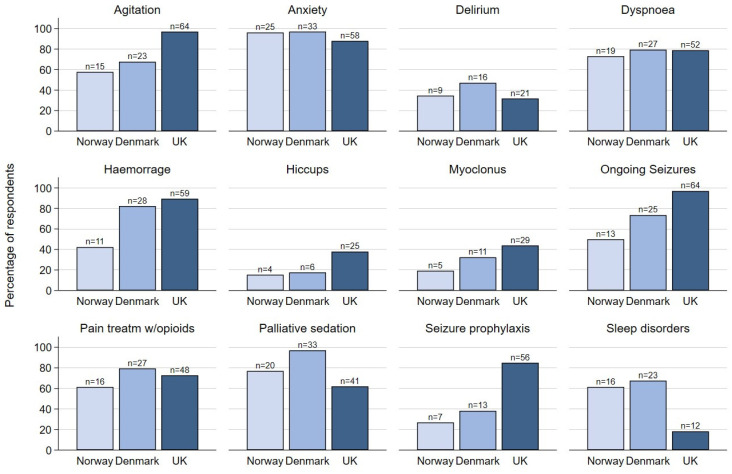
Number and percentage of participants reporting different indications for midazolam in palliative care in Norway, Denmark, and the United Kingdom (UK).

Details on midazolam indications are reported in Figure 1. The most frequently reported indications for midazolam were anxiety (92%), ongoing seizures (81%), agitation (81%), dyspnoea (78%), haemorrhage (78%), palliative sedation (74%), and analgesia in combination with opioids (72%). 

For the indications of anxiety and dyspnoea, there were only minor national differences. Also, for midazolam as an analgesic adjuvant, the responses were comparable. There were, however, national differences for the indications of agitation, seizures, palliative sedation, and sleep disorders. For agitation, UK practitioners more often considered indications for midazolam (NO 68%, DK 58%, UK 97%). Also, for seizure prophylaxis and treatment, UK clinicians more often considered indications for midazolam (NO 27%, DK 38%, UK 85%, and NO 50%, DK 74%, UK 97%, respectively). For palliative sedation, Danish physicians more often considered indications for midazolam (NO 77%, DK 97%, UK 62%), and for sleep disorders, Danish and Norwegian physicians, to a larger extent, considered indications for midazolam than their UK colleagues (NO 62%, DK 68%, UK 18%). For all three countries, relatively few respondents reported midazolam being indicated for hiccups, myoclonus, and delirium.

### 3.3. Midazolam Dosing and Factors Influencing Starting Dose

Details on midazolam starting doses are displayed in Table 2. The response options consisted of predefined starting dose intervals for different indications, and the medians are presented as such. For all indications but palliative sedation, UK respondents reported the highest median CSCI midazolam starting doses. For palliative sedation, Norwegian respondents reported the highest median CSCI midazolam starting dose.

Details on midazolam maximum doses are presented in Table 3. For anxiety, sleep disorders, and haemorrhage, the national responses were comparable with respect to median maximum doses. For the remaining indications, UK respondents reported the highest median maximum doses.

The following factors were assessed for their impact on the midazolam starting dose: indication, body mass index, age, liver function, kidney function, local guidelines, symptom intensity, and prognosis. With some national variations, symptom intensity and indication were the two single factors with the most impact on the midazolam starting dose. Eighty-six percent of the respondents always or frequently considered symptom intensity when deciding on the midazolam starting dose (NO 72%, DK 88%, UK 91%). Eighty-three percent of the respondents always or frequently took indication into account when deciding on the midazolam starting dose (NO 65%, DK 85%, UK 89%). With notable national differences, kidney and liver function were the two single factors with the least impact on the midazolam starting dose. Forty-six percent of the respondents rarely or never considered kidney function when deciding on the midazolam starting dose (NO 84%, DK 78%, UK 17%), and forty-one percent rarely or never took liver function into account (NO 80%, DK 59%, UK 17%).

### 3.4. Factors Influencing Midazolam Discontinuation

The following factors were assessed for their impact on midazolam discontinuation: loss of indication, dependency, adverse events, and lack of effect. All three countries reported a loss of indication as the most impactful factor on midazolam discontinuation, where 56% of all respondents frequently or always considered a loss of indication when discontinuing midazolam (NO 50%, DK 63%, UK 53%). Dependency was reported as the least impactful factor, with 80% of all respondents rarely or never considering dependency as a factor for discontinuing midazolam (NO 80%, DK 75%, UK 83%). In addition, 42% of the respondents rarely or never discontinued midazolam due to adverse events (NO 56%, 58%, UK 32%).

### 3.5. Administration

With notable national variations, most respondents preferred a combination of CSCI and PRN administration for the following indications: midazolam as an analgesic adjuvant: 66% (NO 71%, DK 38%, UK 79%); dyspnoea: 63% (NO 67%, DK 28%, UK 83%); and anxiety: 58% (NO 65%, DK 33%, UK 68%). However, the Danish physicians preferred PRN administration for analgesia (62%), dyspnoea (72%), and anxiety (67%).

### 3.6. Combinations of Midazolam and Other Drugs

The respondents indicated whether they prescribed midazolam as a single-drug or mixed-drug infusion when administered continuously. Few clinicians outside Denmark administered midazolam as a single-drug infusion. Opioids were most frequently the drug combined with midazolam in mixed drug infusions by the participants, where 94% reported they used this combination frequently, followed by the combination of midazolam and haloperidol (47%). 

The least applied drug combination was midazolam and ketamine/s-ketamine; 44% of the respondents never used this combination. There were national variations: 62% of the UK clinicians never combined midazolam and ketamine/s-ketamine, while the corresponding Norwegian and Danish responses were 17% and 20%, respectively. On the other hand, 66% of the UK clinicians frequently combined midazolam and anticholinergic drugs. The corresponding Norwegian and Danish responses were 0% and 11%, respectively.

### 3.7. Treatment Duration and Midazolam after Discharge

For most indications, the physicians reported no time frame limitations for midazolam treatment. Fifty-eight percent of the Norwegian respondents frequently discharged patients with midazolam. The corresponding Danish and UK responses were 10% and 42%, respectively. In addition, whereas 64% of the Danish physicians preferred PRN administration of midazolam at discharge, the corresponding Norwegian and UK responses were 17% and 5%, respectively.

## 4. Discussion

We surveyed midazolam prescription practices among palliative care physicians in NO, DK, and the UK. Midazolam was often used for anxiety, dyspnoea, and as an analgesic adjuvant, but seldom for delirium. Symptom intensity was often decisive for dose increments, and a loss of indication was decisive for midazolam discontinuation. In addition, in all three countries, palliative care physicians frequently combined opioids and haloperidol with midazolam in mixed-drug infusions. Norwegian clinicians used the highest midazolam starting doses for palliative sedation and the highest maximum doses for sleep disorders. Danish clinicians preferred a PRN administration, regularly refrained from midazolam drug admixtures, and were less inclined to use midazolam in outpatient settings. In general, UK clinicians applied the highest midazolam doses and more often considered liver and renal function when deciding on dosing. 

### 4.1. Research and Implementation in Palliative Care 

Palliative care research is methodologically challenging [27], and some researchers argue that this patient population is too fragile, vulnerable, and heterogeneous to allow for valid research [28]. Thus, scientific stringency may be difficult, and clinical practice is often experience-based [29]. There is a deficiency in research on medical treatment options, and even for a defined focus area such as pain treatment, a Cochrane review stated that the quality of evidence for opioids is low [30,31]. In addition, insufficient implementation of new knowledge into practice may impede symptom management in accordance with the available evidence [32]. Hence, both an insufficient evidence base and suboptimal implementation strategies may result in non-beneficial interventions [33]. Furthermore, small studies, perhaps with inferior study designs, conducted on poorly defined subpopulations make generalization of their study results and translation of research into practice even more difficult [34]. A practice survey, like the current one, may disclose heterogeneous practices, management that perhaps should be abandoned, and practices that ought to be studied in clinical trials.

### 4.2. Indications 

Our survey demonstrated both heterogeneous and scientifically unsupported practices. Despite the limited evidence for the drug treatment of anxiety in palliative care patients [6,14], anxiety was the most common indication for midazolam in all three countries. In addition, notwithstanding both available clinical studies and guideline recommendations for the use of midazolam for palliative sedation [20,35], substantial national differences were disclosed. Furthermore, even though the role of midazolam in dyspnoea is debated [17,36], the drug was frequently used for this indication in all three countries. Moreover, the role of midazolam as an adjuvant analgesic is at best based on indirect evidence [15,16,36,37]. Still, the widespread use of midazolam as an analgesic adjuvant was disclosed. And even though midazolam is the preferred drug of choice for ongoing seizures in palliative care, our survey demonstrated substantial national differences [19]. Hence, further studies on midazolam indications in palliative care are needed to provide firm clinical guidance. 

### 4.3. Dosing

In Norway, there is limited available dosing guidance for midazolam in palliative care. One official source provides single-dose recommendations for anxiety, agitation, dyspnoea, and delirium [38], and a regional handbook in palliative care provides CSCI dose recommendations for anxiety and dyspnoea [39]. And even though Norwegian and UK recommendations on midazolam for anxiety and dyspnoea are similar, UK palliative care physicians use higher midazolam starting doses than physicians in Norway and Denmark [24,38,39]. The lack of internationally accepted guidelines may promote diverging practices. Except for palliative sedation and sleep disorders, UK palliative care physicians in general applied the highest midazolam doses. The long history of palliative care in the UK and more familiarity with higher doses may serve as explanations for practice variations. Another possible explanation is national differences in the definitions of palliative sedation and end-of-life care. Since no midazolam studies provide indisputable evidence of time to symptom control, the degree of symptom control, and dosing-related side effects, research addressing these issues in awake palliative care patients is warranted.

### 4.4. Factors Influencing Midazolam Dosing and Discontinuation

Patient-reported symptom intensities are crucial triggers for clinical interventions in palliative care, and symptom intensity was the most important factor for the initial midazolam dose [40]. Indication was the second most important factor for the initial midazolam dose. Still, many physicians used the same start-up dose irrespective of indication. 

Overall, liver and kidney functions were the least considered factors when choosing the starting dose. The fact that UK physicians were most inclined to factor in organ function might be both dependent on initial dosing and how late in the disease trajectory midazolam was introduced. Both reduced renal and liver function may increase midazolam-related sedation [8,41]. The survey provided no answers on the importance of and focus on midazolam side effects in palliative care patients, where separating side effects from symptoms of progressive disease may also be difficult.

Dependency was reported as the least considered factor when discontinuing midazolam. This may indicate that palliative care physicians, to a low degree, emphasize drug dependency issues; however, the survey did not address patient prognosis at midazolam initiation. Late in the palliative care trajectory, midazolam dependency may not be a matter of concern or influence life expectancy [7]. However, with the introduction of early and integrated palliative care services, drug dependency may represent a problem that needs to be addressed [42].

### 4.5. Administration

Norwegian and UK physicians often used a combination of CSCI and PRN, while Danish clinicians regularly preferred on-demand midazolam administration. No studies support either administration mode, but a Danish palliative care textbook’s recommendation on PRN midazolam administration may support the reported practice variations [43]. From a pharmacological point of view, the short half-life of midazolam and sustained duration of symptoms might favour prolonged or continuous treatment over on-demand bolus doses [9]. 

### 4.6. Midazolam in Mixed-Drug Infusions

Opioids and midazolam drug combinations were preferred across all three countries. Given the prevalence of pain in palliative care [4], patients prescribed midazolam may already be treated with opioids, and combination therapy will ease the delivery. Additionally, compatibility studies have demonstrated that the combination of opiates and midazolam is stable in syringe drivers used for continuous infusion [44]. The ketamine/s-ketamine and midazolam combination was the least preferred drug combination. However, Norwegian and Danish physicians seemed more inclined to combine ketamine with midazolam than their British colleagues. Current studies on ketamine in palliative care support the restrictive use of this drug [45]. Still, the study findings indicate that the role of ketamine/esketamine in palliative care should be further investigated.

### 4.7. Treatment Duration and Midazolam after Discharge

Midazolam is often reserved for patients with advanced disease, distressing symptoms, and short expected survival [46], and the survey participants agreed that midazolam was often continued once started. Danish physicians seldom used midazolam after hospital discharge, in contrast to Norwegian and UK physicians. Taking the differences in preferred administration into account, the on-demand use of midazolam may be more convenient to discontinue at discharge compared to continuous administration of the drug.

### 4.8. Strengths and Limitations 

The low response rate is a significant study limitation. However, most of the respondents were experienced palliative care physicians. The survey had a quantitative design yet aimed to address treatment details on the use of midazolam. More possibilities for individualized responses might have better facilitated goal achievement. The small sample size limits the generalizability of the findings.

Another aspect is the patient population in the three surveyed countries. In Norway and Denmark, palliative care patients often have cancer [47], whereas in the UK, palliative care, to a higher degree, includes patients with non-malignant diagnoses [48]. 

National differences in the use of terminology may also have affected the results [4,5]. This point may apply to both symptoms like anxiety and agitation and interventions like palliative sedation. In addition, palliative populations are heterogeneous, with large variations in expected survival. Opting for multiple responses and reservations may have provided deeper insight. As the survey did not address these issues, the yielded responses must be interpreted with caution.

The survey was conducted in NO, DK, and the UK. These three countries were selected due to the availability of corroborators for the research group and, as such, recruitment to the survey. The selection is not representative of the whole of Europe and must be interpreted as such. However, the results indicate national differences.

The survey did not address the settings for midazolam administration and thus provides no information on potential practice differences at different levels of the health care system.

### 4.9. Future Perspectives

With the lack of consensus on the use of midazolam in palliative care, future research should address for which indications midazolam treatment should be initiated and the optimal dosing of midazolam for these indications to provide more standardized use of midazolam in palliative care. Further research into the co-administration of drugs with midazolam in admixtures and their compatibility to ensure patient safety is also warranted. 

## 5. Conclusions

This survey described national variations in the off-label use of midazolam in palliative care. Practices in Norway, Denmark, and the UK were similar regarding the use of midazolam for anxiety, dyspnoea, delirium, and pain treatment in combination with opioids. Often, UK physicians used higher midazolam doses compared to physicians in Norway and Denmark. Danish physicians more often used on-demand midazolam administration compared to a combination of CSCI and PRN in the other two countries. In addition, we disclosed national differences in syringe driver drug combinations.

## Figures and Tables

**Table 1:** Survey participant characteristics.

Characteristics	Norway (NO)		Denmark (DK)		United Kingdom (UK)		Total
No of participants, N (%)	26	(21)	34	(27)	66	(52)	126
Level of experience, N (%)							
Junior doctor	0	(0)	0	(0)	2	(3)	2
Resident	1	(4)	1	(3)	2	(3)	4
Senior doctor	12	(46)	9	(26)	5	(8)	26
Specialist in Palliative Medicine	13	(50)	24	(71)	57	(86)	94
Years of experience in palliative care, N (%)							
Up to 1 year	0	(0)	2	(6)	1	(1)	3
1–5 years	6	(23)	12	(35)	13	(20)	31
6–10 years	8	(31)	12	(35)	11	(17)	31
More than 10 years	12	(46)	8	(24)	41	(62)	61

**Table 2:** Median midazolam starting doses (mg/24 h, continuous subcutaneous infusion) for different indications.

Indication	n *	Median	Indication	n *	Median
Anxiety			Myoclonus		
NO	20	3.1–5.0	NO	3	5.1–10.0
DK	21	3.1–5.0	DK	6	5.1–10.0
UK	55	5.1–10.0	UK	25	5.1–10.0
Dyspnoea			Agitation		
NO	17	3.1–5.0	NO	12	3.1–10.0
DK	14	3.1–5.0	DK	12	3.1–5.0
UK	51	5.1–10.0	UK	63	5.1–10.0
Seizure prophylaxis			Delirium		
NO	6	5.1–10.0	NO	7	3.1–5.0
DK	6	5.1–10.0	DK	8	2.1–5.0
UK	32	10.1–20.0	UK	19	5.1–10.0
Ongoing seizures			Sleep disorders		
NO	5	5.1–10.0	NO	12	3.1–10.0
DK	8	5.1–10.0	DK	9	3.1–5.0
UK	15	10.1–20.0	UK	4	3.1–5.0
Hiccups			Palliative sedation		
NO	3	3.1–5.0	NO	17	15.1–20.0
DK	4	1.1–2.0	DK	22	10.1–20.0
UK	23	10.1–20.0	UK	36	10.1–20.0
Haemorrhage			Pain treatment in combination with opioids		
NO	4	5.1–20.0	NO	14	3.1–5.0
DK	11	10.1–20.0	DK	17	3.1–5.0
UK	16	10.1–20.0	UK	47	5.1–10.0

* Only respondents who stated that midazolam was indicated for the condition were able to suggest dosing.

**Table 3:** Median midazolam max. doses (mg/24 h, continuous subcutaneous infusion) for different indications.

	n *	Median (IQR)		n *	Median (IQR)
Anxiety			Myoclonus		
NO	19	20 (10–25)	NO	5	10 (10–20)
DK	20	15 (10–22)	DK	8	12.5 (5–20)
UK	51	20 (15–50)	UK	22	20 (10–30)
Dyspnoea			Agitation		
NO	16	15 (10–25)	NO	10	20 (5–20)
DK	17	15 (5–20)	DK	12	20 (9–35)
UK	46	30 (20–30)	UK	60	60 (55–100)
Seizure prophylaxis			Delirium		
NO	6	25 (20–30)	NO	7	20 (15–30)
DK	6	15 (2.5–30)	DK	8	10 (5–30)
UK	50	30 (30–50)	UK	20	45 (25–60)
Ongoing seizures			Sleep disorders		
NO	9	10 (5–30)	NO	11	10 (3–20)
DK	14	20 (10–30)	DK	14	5 (5–20)
UK	59	60 (50–90)	UK	12	10 (7.5–20)
Hiccups			Palliative sedation		
NO	3	10 (7.5–20)	NO	15	50 (20–100)
DK	3	5 (5–5)	DK	18	50 (20–80)
UK	22	20 (10–30)	UK	35	80 (60–100)
Haemorrhage			Pain treatment in combination with opioids		
NO	8	10 (5–30)	NO	12	15 (5–25)
DK	16	17.5 (10–35)	DK	18	10 (5–20)
UK	51	10 (10–40)	UK	43	30 (20–30)

* Only respondents who stated that midazolam was indicated for the condition were able to suggest dosing.

## Data Availability

The authors confirm that the data supporting the findings of this study are available within the article. Any other information supporting the findings is available from the corresponding author (MT) upon reasonable request.
